# Human intestinal lipid storage through sequential meals reveals faster dinner appearance is associated with hyperlipidemia

**DOI:** 10.1172/jci.insight.148378

**Published:** 2021-08-09

**Authors:** Miriam Jacome-Sosa, Qiong Hu, Camila M. Manrique-Acevedo, Robert D. Phair, Elizabeth J. Parks

**Affiliations:** 1 Department of Nutrition and Exercise Physiology and; 2 Division of Endocrinology, School of Medicine, University of Missouri, Columbia, Missouri, USA.; 3 Integrative Bioinformatics, Inc., Mountain View, California, USA.; 4 Division of Gastroenterology and Hepatology, School of Medicine, University of Missouri, Columbia, Missouri, USA.

**Keywords:** Metabolism, Insulin, Lipoproteins

## Abstract

**Background:**

It is increasingly recognized that intestinal cells can store lipids after a meal, yet the effect of this phenomenon on lipid absorption patterns in insulin resistance remains unknown.

**Methods:**

The kinetics of meal fat appearance were measured in insulin-sensitive (IS, *n* = 8) and insulin-resistant (IR, *n* = 8) subjects after sequential, isotopically labeled lunch and dinner meals. Plasma dynamics on triacylglycerol-rich (TAG-rich) lipoproteins and plasma hormones were analyzed using a nonlinear, non–steady state kinetic model.

**Results:**

At the onset of dinner, IS subjects showed an abrupt plasma appearance of lunch lipid consistent with the “second-meal effect,” followed by slower appearance of dinner fat in plasma, resulting in reduced accumulation of dinner TAG of 48% compared with lunch. By contrast, IR subjects exhibited faster meal TAG appearance rates after both lunch and dinner. This effect of lower enterocyte storage between meals was associated with greater nocturnal and next-morning hyperlipidemia. The biochemical data and the kinetic analysis of second-meal effect dynamics are consistent with rapid secretion of stored TAG bypassing lipolysis and resynthesis. In addition, the data are consistent with a role for the diurnal pattern of plasma leptin in regulating the processing of dietary lipid.

**Conclusion:**

These data support the concept that intestinal lipid storage may be physiologically beneficial in IS subjects.

**Trial registration:**

ClinicalTrials.gov NCT02020343.

**Funding:**

This study was supported by a grant from the American Diabetes Association (grant 1-13-TS-12).

## Graphical abstract

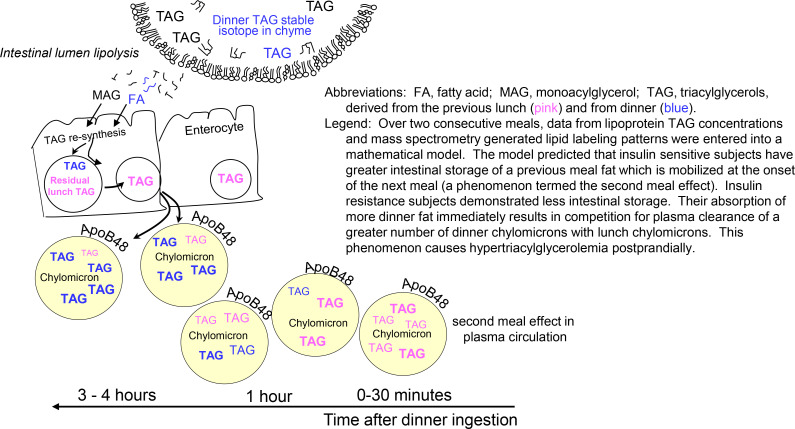

## Introduction

High plasma concentrations of postprandial triacylglycerols (TAGs) are an independent risk factor for cardiovascular disease (1, 2) and a key clinical feature of atherogenic dyslipidemia in insulin resistance (3). The association of elevated postprandial TAGs with heart disease risk is the strongest when TAGs are measured approximately 4 to 6 hours postprandially (4–6). Insulin resistance is central both to the overproduction of TAG-rich lipoproteins (TRLs), including hepatic-derived VLDL and intestinally derived chylomicrons (7–10) and to the slowed clearance of these particles during the postprandial state (11, 12). Over the past decades, research has demonstrated that the natural circadian pattern of plasma TAG concentrations is characterized by a steady rise in levels throughout the day, with peak concentrations occurring in the middle of the night (13–16). Thus, chylomicrons made at breakfast are still circulating when lunch-chylomicrons enter the blood, and dinner-chylomicrons further add to the plasma lipid burden late in the day. Another consistent observation is that when research participants were fed 2 fat-containing meals (e.g., breakfast and lunch), consumption of the second meal elicited a very early increase in plasma TRL-TAG, occurring before the second meal could have moved through the intestine. This phenomenon, referred to as the second-meal effect (SME) by Jackson and colleagues (17), has been observed many times (18–21, 22), and its existence suggests that the intestine has a substantial capacity to store fat. Elegant cell biology studies and rodent research have significantly increased the understanding of intestinal lipid handling (23–33). Studies in humans, however, have been limited by the inability to sample intestinal lipid pools in people, except through single time-point biopsies (34, 35). The present study utilized isotopic labeling of meals to track the presence of meal fat in the blood over a 19-hour period. This was followed by nonlinear, non–steady state kinetic modeling of the meal lipid’s appearance in plasma TRL-TAG and nonesterified fatty acids (NEFAs) of insulin-sensitive (IS) subjects and insulin-resistant (IR) subjects. The primary goals of this investigation were to quantitate the impact of insulin resistance on the processes of lipid absorption and test the hypothesis that intestinal fat storage contributes to the SME in humans independent of intracellular lipase-mediated lipolysis. Given that IR subjects have excess TAG stored in the liver (36) and skeletal muscle (37), we hypothesized that these subjects would also exhibit increased fat storage in the intestine. Surprisingly, the present data support an opposite conclusion — that intestinal lipid storage is limited in insulin resistance, resulting in more rapid transfer of lipid into plasma, particularly after dinner, when the diurnal pattern of plasma TAG is at its peak. Findings also support a role for leptin in the reduced appearance of dinner fat compared with lunch, consistent with leptin-mediated regulation of fatty acid oxidation (38), intestinal and hepatic lipid handling (39–41), and postprandial TAG excursions (42) tested in vitro and in mice. These data indicate that the normal physiological response to sequential meal consumption in healthy subjects includes slowed lipid transport later in the evening. If this is the case, strategies can be developed to treat the dyslipidemia of insulin resistance to reduce nocturnal lipemia and lessen the atherogenicity of the postprandial state.

## Results

**Figure 1:**
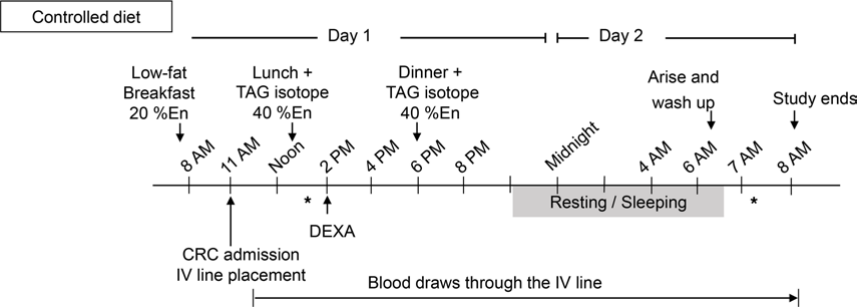
Study protocol. The inpatient study day was preceded by a 3-day isoenergetic controlled diet. Isotope-labeled meals of identical composition were served at 12:30 pm and 6:00 pm. %En, percentage of the subject’s daily energy needs; * indicates the timing of indirect calorimetry.

Subjects without diabetes were grouped based on insulin sensitivity (S_i_) measured during the frequently sampled, insulin-modified, intravenous glucose tolerance test (FS-IVGTT), and the groups were matched for sex and age (Table 1). The study design is shown in Figure 1, the flow of study participants in Supplemental Figure 1 (supplemental material available online with this article; https://doi.org/10.1172/jci.insight.148378DS1), and the composition of the lunch and dinner research meals in Table 2. Subjects participated in an overnight inpatient study to measure the kinetics of dietary fat appearance using isotopically labeled lunch and dinner meals of identical composition (see Methods), followed by mass spectrometry of plasma lipid and nonlinear, non–steady state kinetic modeling (described below). Compared with IS subjects, IR subjects had higher BMIs, body weights, and fat mass (Table 1). Except for expected higher insulin and Homeostatic Model Assessment for Insulin Resistance in IR subjects, the IS and IR subjects were comparable in their acute insulin response to glucose (AIRg), disposition indexes, and fasting plasma biochemistries. It is possible that the lack of difference in some of the outcomes was due to the small sample size. Baseline ad libitum dietary intakes were similar between groups (Table 2). Fasting and fed-state total energy expenditure did not differ between groups (Table 1), although IR subjects had approximately 53% lower fasting and fed-state glucose oxidation (*P* = 0.009) and 42% higher fed-state fat oxidation compared with IS subjects (*P* = 0.042).

### Plasma insulin, TRL-TAG sources, and the SME.

**Figure 2:**
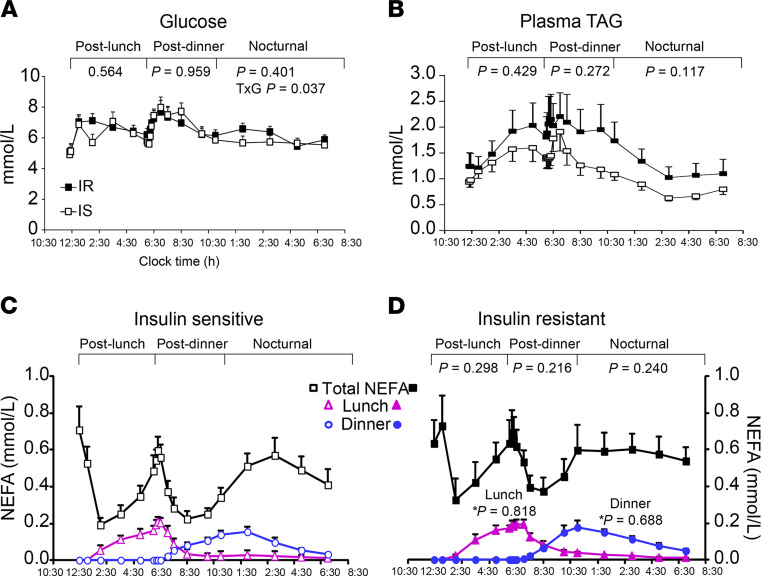
Glucose, plasma TAG, NEFA, and NEFA sources in IS and IR subjects. Values represent the mean ± SEM. Data were divided into 3 segments for analysis: 12:00 pm to 6:00 pm (post-lunch), 6:00 pm to 11:00 pm (post-dinner), and 11:00 pm to 7:00 am (nocturnal). (**A**) Plasma glucose and (**B**) triglyceride concentrations in IS (open squares) and IR (filled squares) subjects. Plasma NEFA sources are presented for subjects who were IS (**C**, open squares) and IR (**D**, filled squares), with lunch NEFAs represented by pink triangle symbols and dinner NEFAs, blue circles. *P* values represent comparisons between the IS and IR groups for glucose, plasma TAG, and total NEFAs for each segment (determined by repeated-measures 2-way ANOVA). For NEFA sources, *P values represent between-group comparisons for lunch and dinner NEFAs throughout the study period.

**Figure 3:**
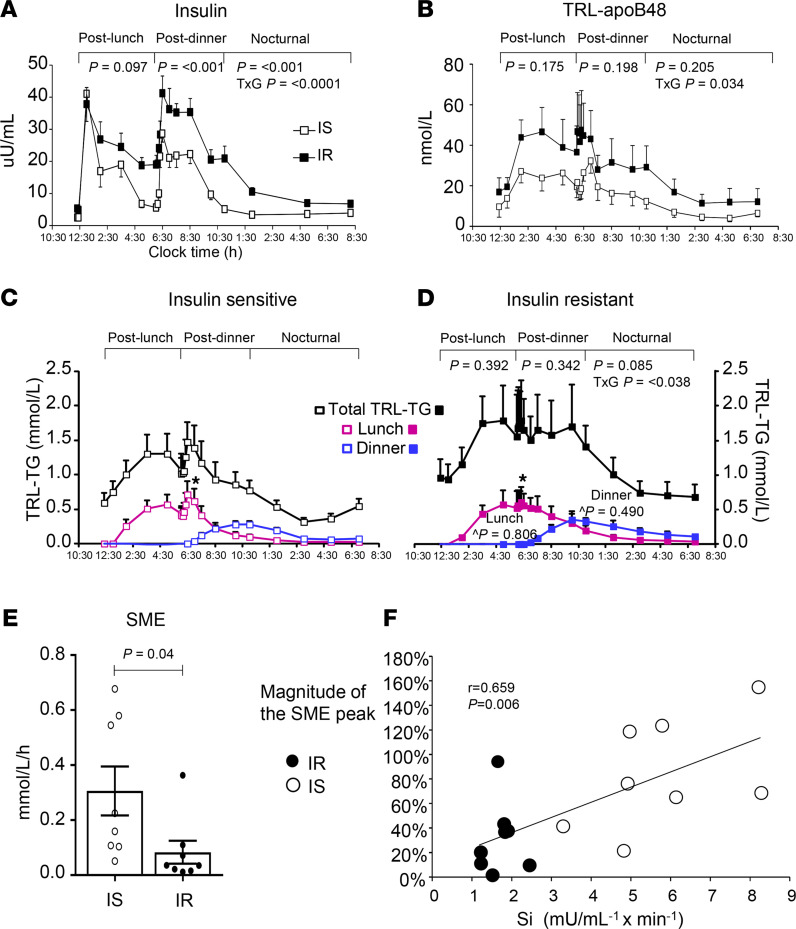
Dynamics of plasma insulin, apoB48, and dietary fat. Values represent the mean ± SEM. (**A**) Plasma insulin concentrations were significantly different between the groups (2-way repeated-measures ANOVA, *P* values for each segment); (**B**) TRL-apoB48 concentrations and (**C** and **D**) total TRL-TAG (black squares) were not significantly different. Interactions among insulin, TRL-apoB48, total TRL-TAG, and subjects’ insulin sensitivity status were statistically significant during the nocturnal period (TxG *P* < 0.05, repeated-measures 2-way ANOVA). TRL-TAG lipid sources are presented for IS (**C**, open squares) and IR subjects (**D**, filled squares), with lunch lipid represented by pink square symbols and dinner lipid, blue squares. ^*P* values represent between-group comparisons for lunch and dinner throughout the study period. (**E**) The second-meal effect (SME) peaks, denoted with asterisks in panels **C** and **D**, were significantly different between the groups when analyzed by absolute AUC. (**F**) The relationship between the insulin sensitivity index (S_i_) and the magnitude of the SME.

**Figure 4:**
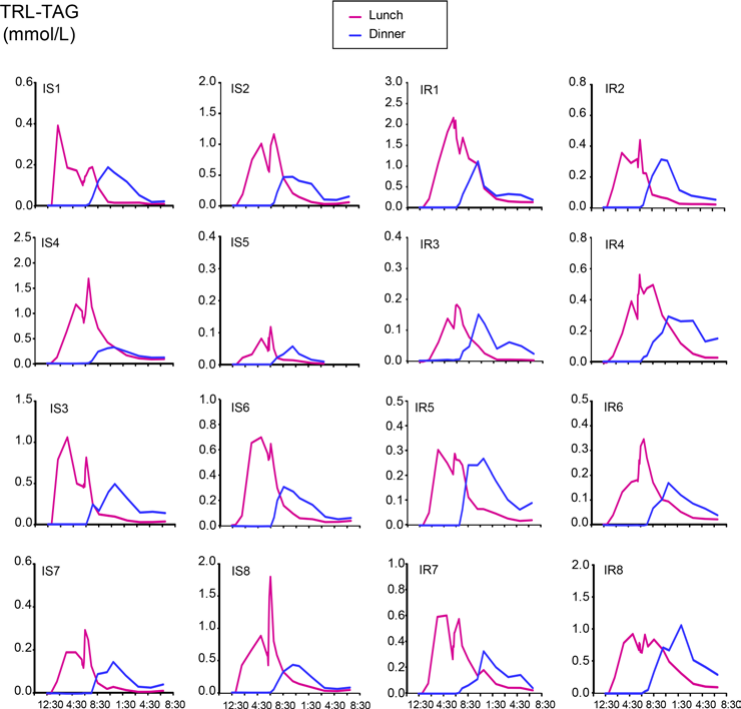
Individual patterns of meal lipid appearance in plasma TRL-TAG. Concentrations of TRL-TAG derived from lunch and dinner in IS and IR subjects. Pink line, TRL-TAG derived from lunch; blue line, TRL-TAG derived from dinner.

Figure 2 shows the similarity between the groups for plasma glucose, TAG, and NEFA concentrations, presented separately for the groups. The diurnal pattern of plasma insulin was significantly higher in the IR subjects (Figure 3A), and these subjects exhibited greater TRL-apoB48 responses, although the difference between the groups was not significant, except for a group × time interaction in the early hours (Figure 3B). The group mean TRL-TAG responses are presented for IS (Figure 3C) and IR subjects (Figure 3D). The dinner-induced SME peaks are denoted with asterisks in Figure 3, C and D. Individual data are presented in Figure 4. When the increases above pre-meal TRL-TAG values were analyzed, the IS subjects’ SME peaks were, on average, 3.7-fold higher than the IR subjects’ peaks (Figure 3E; *P* = 0.04). The greater the S_i_, the greater the SME peak, calculated as a percentage of that above the expected decay of the label after lunch (*r* = 0.659, *P* = 0.006; Figure 3F). This concept was further supported by kinetic analysis using the model of lipid absorption illustrated in Supplemental Figure 2. Details of the modeling process are presented in the Methods, and the model was implemented as a system of nonlinear ordinary differential equations. The model, built based on published data, including kinetic values for enzymatic activities as performed previously (43), could resolve chylomicron kinetics, even though we analyzed the TRL fraction, which also contains VLDL. This is possible because the SME provides a near-bolus of chylomicron label delivered directly to plasma. Table 3 presents the results, and the computed mass of stored TAG released as SME trended to be higher in IS subjects (row 5, *P* = 0.056). With respect to the chylomicron TAG-palmitate secretion rate, this was 36% greater in IS compared with IR subjects (row 6, *P* = 0.056), although this was countered by an 81% greater chylomicron TAG-palmitate FCR in IS subjects (row 7, *P* = 0.052). The net result accounted for the greater plasma accumulation of dietary fat in IR subjects.

### Differing rates of absorption of lunch fat versus dinner fat.

**Figure 5:**
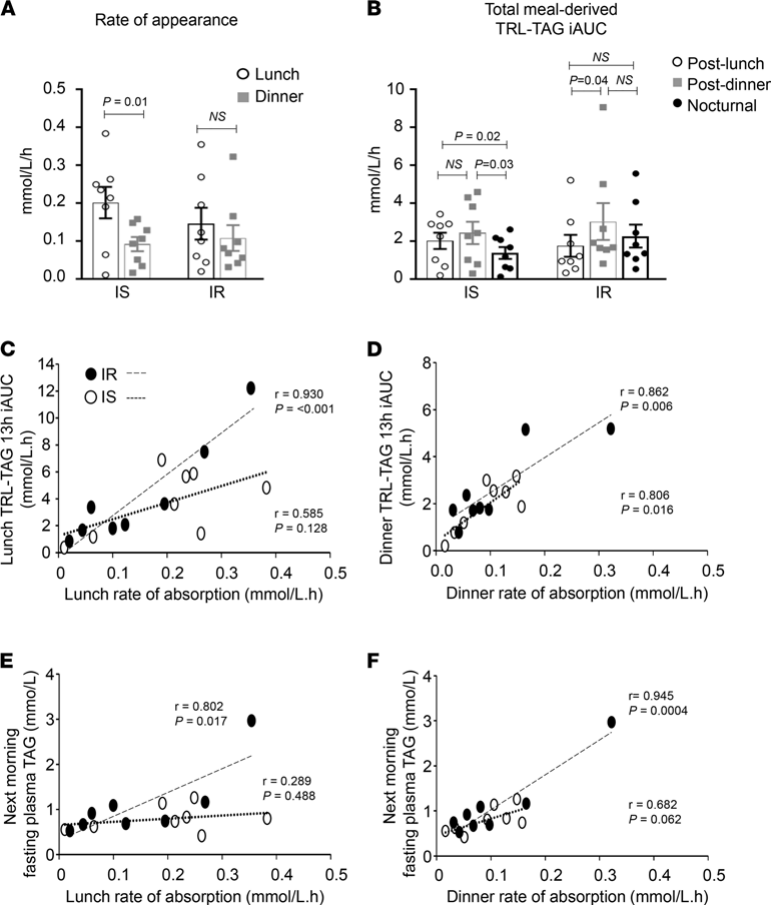
Meal appearance rate in plasma, the presence of meal label, and influence of meal rates of appearance on TRL- and plasma-TAG. Values represent the mean ± SEM. (**A**) IS subjects demonstrated a slower rate of appearance of dinner lipid in plasma (within-group comparison, paired, 2-tailed *t* test). (**B**) Total meal-derived lipid was higher after dinner in IR subjects (within-group comparison over time, repeated-measures 1-way ANOVA) and lower nocturnally in IS subjects. Relationships between rates of appearance of meal lipid and meal-derived TRL-TAG incremental AUC for (**C**) lunch and (**D**) dinner. Relationships between rates of appearance of meal lipid and the fasting TAG the next morning for (**E**) lunch and (**F**) dinner.

We compared post-lunch and post-dinner lipemic responses and found that for IS subjects, dietary fat appearance rate after dinner was slower than after lunch (Figure 5A). In a separate analysis (Figure 5B), we compared the presence of lunch lipid in plasma during the 5-hour period after lunch (post-lunch), the combination of lunch and dinner lipid together in plasma after dinner (post-dinner), and the combination of lunch and dinner lipid remaining in plasma throughout the night (nocturnal). Throughout the nocturnal phase, IS subjects exhibited a 31%–43% reduction in meal lipid derived from the sum of lunch and dinner in TRL-TAG. By contrast, in IR subjects, meal lipid was 1.7-fold higher in the post-dinner period relative to the post-lunch period, and this remained elevated through the nocturnal phase (Figure 5B). For IR, a significant positive association was observed between the plasma accumulation rates of lunch (Figure 5C) and the persistence of this lipid in plasma (the faster the accumulation, the longer the label was detected in TRL). After dinner, this relationship was significant for IR and IS subjects (Figure 5D). In IR subjects, the accumulation rate also correlated with fasting TAG concentrations the next morning, although both relationships were weaker or absent in the IS subjects (Figure 5, E and F).

### The diurnal leptin amplitude and lower evening meal hypertriglyceridemia.

**Figure 6:**
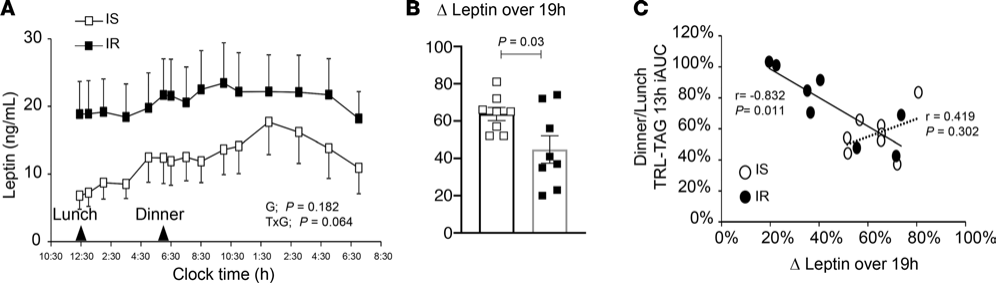
Influence of leptin on meal rates of appearance. (**A**) The interaction between subjects’ insulin sensitivity status and leptin concentrations over time tended to be significant (TxG; *P* *=* 0.064). (**B**) IS subjects exhibited a greater change in leptin over the 19-hour period. (**C**) The relationship between the change in leptin over 19 hours and the ratio of dinner to lunch TRL-TAG.

One striking feature of the lunch and dinner tracer data (Figure 3, C and D) was the lesser plasma accumulation (AUC) of dinner fat compared with lunch fat despite identical meal composition, quantity, and energy content relative to total energy needs. We tested several mechanistic hypotheses to explain the reduced dinner fat appearance, and the results from the model did not reveal a regulation of this phenomenon by insulin. We next tested the effects of the hormone leptin because dietary manipulations that elevate plasma TAG concentrations (high fat or high fructose) are associated with smaller leptin pulse amplitudes (e.g., smaller absolute increases in leptin from nadir to peak over 24 hours). In the present study, the change in leptin concentrations over the 19-hour protocol were lower in IR than IS subjects (Figure 6, A and B). IR subjects exhibited higher pre-lunch leptin concentrations (18.9 ± 13.7 ng/mL), which remained constant throughout the day. However, within the IR group, the diurnal rise in leptin was variable between subjects, and individual leptin pulse amplitudes were negatively correlated with the ratio of dinner AUC to lunch AUC (*r* = –0.832, *P* = 0.011; Figure 6C). This means that in IR subjects, the flatter the leptin curve, the greater the appearance of dinner lipid in plasma and the longer its circulation compared with lunch.

### When modeling intra-enterocyte events, SME kinetics suggest fusion of cytosolic lipid droplets to enterocyte ER.

Rapid appearance in the blood of a bolus of lunch fat upon eating or even anticipating dinner, which we refer to as the SME, suggests that the processes occurring between enterocytes and subclavian vein are fast on the time scale of the experiment. By contrast, the appearance of the bulk of meal fat in the blood is slow. According to the model, the further inference is that the processes between acyl-CoA and resynthesized TAGs (Supplemental Figure 2), represented in the model by esterification through an enzyme like diacylglycerol acyltransferase (DGAT) (shown in Supplemental Figure 2, process 2), are rate limiting. Values obtained for the esterification rate constant (Table 3, row 2) trended higher for the IS than the IR group.

Having calculated esterification kinetics through modeling, the data suggested that hydrolysis of lipid droplet (LD) TAG, no matter how rapid, could not account for the SME. Resynthesis of hydrolyzed fatty acids into TAGs and immediate assembly and secretion as chylomicrons would be as slow to appear in plasma as the bulk of lunch lipid between 12:30 pm and 4:30 pm in Figure 3C, for example. Consequently, the hydrolysis-resynthesis model was too slow and completely failed to account for the rapid kinetics of SME production. If this is correct, the SME consists of preformed TAGs, and thus the data are consistent with a model in which enterocyte cytosolic LDs fuse with the ER membrane in response to a neuroendocrine signal (Supplemental Figure 2, process 8). These TAGs may have been diverted from the ER “lens” pool (32) by budding of LDs during the previous meal. The model predicted that at least 25% of esterification-produced, resynthesized TAGs traversed this pathway (Table 3, row 3), and 75% was loaded directly onto nascent chylomicron particles (Table 3, row 4) for secretion into lymph and transport to plasma. These calculated parameters were similar between the groups. The fusion model has the advantage that it can account simultaneously for observations of postprandial LD accumulation in enterocytes and one of the most reproducible and distinctive features of successive meals, the pulse of TRL-TAG referred to as the SME. According to the model, when the SME peak was compared with the total lipid in the lunch meal, 33.6% ± 22.9% of lunch lipid was held in the intestine between lunch and dinner (data not shown). Other model-generated hypotheses can be advanced and tested in future research. The enterocyte LD-TAG that is released by dinner as SME (e.g., row 5) was predicted to be 2-fold higher in IS subjects (51.5 ± 28.1 μmol/kg BW) compared with IR subjects (26.0 ± 16.5 μmol/kg BW, *P* = 0.056). A principal reason for this requirement is the faster removal of plasma chylomicron TAG in IS subjects (Table 3, rows 7–9). The dynamics of the SME isotopic data yield information about chylomicron metabolism that would otherwise be unavailable in TRL-TAG kinetic data.

### Correlations suggest further mechanistic interpretations.

As described above, the final kinetic model included parameters that are listed in Table 3. Model parameters are echoed in a number of physiological relationships generated independently by AUC and statistical analysis. First, the faster the meal TAGs were absorbed into the intestine, the greater the S_i_ (Supplemental Figure 3A) and interestingly, the lower the ratio of dinner to lunch peak (Supplemental Figure 3B). However, these relationships were driven by data from the IS subjects. Second, for all subjects combined, the greater the predicted fraction of lipid moving to intestinal LDs via esterification, the lower the rate of lunch label movement into the plasma (Supplemental Figure 3C). Third, the SME was related both to greater S_i_ (Supplemental Figure 3D) and faster meal TAG absorption in the intestine (Supplemental Figure 3E). Interestingly, the greater the SME, the lower the ratio of dinner to lunch peak (Supplemental Figure 3F). Last, with regard to chylomicron-TAG clearance, the fraction of TAG palmitate being taken up directly (Table 3, row 12) tended to be higher in IS subjects, while remnant production (row 13) did not differ; neither did the spillover of chylomicron-TAG into the plasma NEFA pool (row 14). As shown in rows 15 and 16, the model predicted that the groups were similar with respect to chylomicron-remnant TAG-palmitate production rate and FCR.

### TAG clearance and NEFA kinetics.

Using the measured dynamics of plasma leptin, we also tested the hypothesis that a diurnal increase in lipid uptake (and therefore decreased lipemia) would be related to adipokine concentrations. Implemented as an activation term (as discussed in the legend of Supplemental Figure 2), this nonlinear mechanism allowed the model to account for decreased accumulation of dinner fat in plasma compared with lunch. The *K_A_* (row 10, *P* = 0.094) trended higher, and the multiplicative factor B (row 11) was different for the 2 groups largely because of the apparent leptin insensitivity of the IR subjects. The phenomenon is the same as can be seen by calculating the ratios of B to *K_A_* (0.14 for IS, 0.15 for IR). Thus, the hypothesis that leptin accelerates chylomicron-TAG uptake is consistent with the experimental data and accounts for a portion of the lower accumulation of dinner fat compared with lunch. Mechanisms that mediate the differential handling of lunch versus dinner may comprise a healthy response to dietary fat.

With regard to NEFA fluxes, the calculated appearance rate of NEFAs from the sum of the lipolytic activities within the adipocyte (row 17) was 2-fold lower in IS compared with IR subjects (3.1 ± 0.7 vs. 6.3 ± 1.7, *P* < 0.001). We have previously demonstrated the importance of insulin to stimulate NEFA uptake into tissues (44), and 3 parameters in the present model characterized this effect of insulin. The activation constant for insulin to double NEFA uptake (row 18) was found to be 2-fold lower in IS subjects compared with IR subjects (*P* < 0.001). In other words, at a lower level of insulin in IS subjects, the hormone increased tissue NEFA uptake. Both the fraction of NEFA flux (row 19, in units of pools/h) and the time-averaged NEFA uptake into peripheral tissues across the day (row 20, μmol/h/kg BW) were significantly lower in IS subjects (*P* = 0.023 and *P* = <0.001, respectively). Plasma NEFA concentrations appeared similar between the groups (Figure 2, C and D), which suggests that the higher IR adipose NEFA release rate was partially compensated for by a hyperinsulinemic postprandial response that stimulated NEFA uptake into tissues.

## Discussion

Although much research has focused on the atherogenicity of hypertriglyceridemia (1, 2, 4, 5), few studies have investigated how enterocyte lipid handling influences postprandial lipid excursions in the physiological setting of eating meals. The present research significantly advances these concepts through the use of isotopically labeled meals, mass spectrometry, and kinetic modeling. Four features of our data stand out. First, insulin sensitivity (increased S_i_) was positively associated with a predicted increase in enterocyte lipid storage — which, if true, suggests that intestinal LDs formed during apical uptake of dietary fat are physiologically desirable. Second, a healthy response to consecutive meals may involve mechanisms that reduce postprandial lipemia by buffering absorption through retention of more of the second-meal lipid in the gut, possibly in response to increasing plasma leptin. Third, we add to the evidence that enterocytic resynthesis of TAGs may be one of the rate-limiting steps between meal consumption and appearance of dietary fat in blood. Fourth, if intestinal esterification is rate limiting in lipid transport, the hypothesis that the SME is caused by stimulated hydrolysis of cytosolic LDs and immediate incorporation of this lipid into nascent chylomicrons would be untenable. The present study contributes in vivo evidence to a long-standing area of lipid cell biology consistent with fusion of enterocyte cytosolic LDs with TAGs in the ER membrane, referred to as the “lens pool” (32). If microsomal triglyceride transfer protein–mediated nascent particle assembly is slow, this fusion may correspond to the second step of lipoprotein assembly where LDs coalesce (in the smooth ER or perhaps the Golgi) with nascent lipoproteins to form the mature lipoprotein particle (45), followed by rapid secretion as new chylomicrons whose lipid derives from the previous meal.

### Insulin resistance and intestinal lipid storage.

Research has provided indirect evidence that insulin sensitivity increases intestinal lipid storage capacity in humans. Fasted jejunal TAG concentrations are higher in controls compared with type 2 diabetics, and jejunal TAG levels are inversely associated with plasma apoB48 and TRL-TAG concentrations (46). Similarly, compared with IR subjects, healthy controls exhibited higher expression of intestinal TAG synthesis genes and an approximately 2-fold lower apoB48 production rate (47, 48). Meal lipid transport was not quantified in these studies, but the sum of these data, along with the present findings, strongly suggest that insulin resistance is associated with more meal lipid being absorbed on greater quantities of apoB48 particles being secreted. Given ectopic lipid deposition in tissues of IR subjects (36, 37), one might assume that intestinal fat storage would have negative metabolic consequences. However, intestinal lipid storage may provide a benefit to health. Numerous studies have shown that in healthy subjects, fatty food consumption throughout a day results in plasma TAG concentrations rising steadily, peaking just after midnight, and falling slowly back to fasting at approximately 4:00 am (14–16). Thus, the potential benefits of intestinal lipid storage include slowing evening chylomicron production to reduce plasma competition of TRL particles for lipoprotein lipase-mediated clearance (49). During fasting, intestinally stored lipid may support constitutive apoB48 particle synthesis (50, 51) to allow for a fast ramp-up of particle production when a bolus of lipid is consumed at the next meal. Intestinal lipid stores also provide fatty acids essential for membrane formation and cellular division and turnover (28) and substrates for oxidation (along with plasma NEFA taken up at the enterocyte basolateral membrane; ref. 26), to fulfill the relatively large energy demands of the intestine — accounting for up to 26% of total body oxygen consumption (52, 53).

### The role of leptin in dietary fat processing.

Leptin, which has also been implicated in the control of lipid metabolism independent of its effects on energy balance (39, 40), exhibits distinct diurnal rhythmicity. Past studies have related diurnal patterns of plasma leptin concentrations with hyperlipidemia (54–59).

Also important in limiting nocturnal lipemia is the rapid removal of chylomicron-TAG. Since plasma leptin tended to increase throughout the 19-hour protocol (Figure 6A), we added to the model the hypothesis that leptin regulates direct chylomicron-TAG uptake (Supplemental Figure 2, process 5) and asked if it could account for the discrepancy. It did so in all subjects, both IS and IR (Table 3, rows 10 and 11). Importantly, it would be equally consistent with the data to move some of this leptin dependence to the enterocytic oxidation or phospholipid synthesis pathway as indicated by the dashed line for leptin control (Supplemental Figure 2, process 1). There is precedent for both leptin control mechanisms (38, 60) and for leptin signaling in the intestine to regulate lipid absorption and plasma TAG (39).

### Quantifying the SME.

We and others have shown that the SME can occur simply in response to tasting food (21, 61), and our laboratory estimated that 25% of the TAG appearing during a morning sensory test was derived from the previous dinner (61). In the present study, SME peaks containing the lunch label were observed in all subjects at the onset of dinner, with plasma areas that ranged from 1% to 155% above that which would have been expected in plasma if dinner had not been fed. The kinetic model predicted approximately 33% of the total lunch lipid entered the blood after the onset of dinner. Originally, we hypothesized that insulin resistance would be associated with greater SME peaks. Unexpectedly, we found the opposite to be true. Indeed, IS subjects exhibited SME peak areas that were 3.7-fold larger than those of the IR subjects, and these lunch peaks in IS subjects were followed by very low levels of dinner lipid in plasma. It is clear the SME is a very fast process (Figure 4). The model has generated a number of hypotheses — one of which is that the SME cannot be produced by rapid cellular hydrolysis of LD-TAG because the released fatty acids would have to traverse resynthesis pathways that are relatively slow. Indeed, the strong inference is that the SME consists of preformed TAGs stored in cytosolic LDs during the previous meal. The model is quantitatively consistent with the hypothesis that in response to meal-induced neurohumoral signals, cytosolic LDs fuse with the ER lens pool and supply chylomicron assembly and secretion. Effectively, the gut loads a biological syringe with endogenously labeled TAGs and rapidly converts them to plasma chylomicrons at the onset of the next meal. An alternative hypothesis was also tested. It has been proposed (62) that an SME could result from smooth muscle contraction in lacteals propelling a bolus of already-secreted chylomicrons toward the lymphatic exit. We implemented this hypothesis by adding a slower (slow enough to accumulate the required bolus) process in the secretory/lymphatic pathway. It was possible to accumulate the required TAGs while still fitting the plasma data. We favor the LD fusion hypothesis for several reasons: it not only fits the plasma data but also provides an account of the well-documented diurnal pattern of enterocytic lipid storage and release (27, 63). Further, the lymph peristalsis mechanism is also not entirely mathematically consistent with the SME labeling pattern. Data from our past and present research showed SME peaks with percentage enrichments greater than or equal to the lunch peak that occurred 2 hours earlier (Figure 4, participants IS 4, IS 8, and IR 6), which are unexplained by the lymph peristalsis hypothesis. Moreover, it appears unlikely that meal-induced lymph peristalsis would be only half as effective in IR subjects. An experiment that could distinguish between these hypotheses is a bolus of labeled leucine administered shortly before the second meal. The LD fusion hypothesis predicts SME chylomicrons are newly synthesized and may be labeled; the lymph peristalsis hypothesis predicts unlabeled SME chylomicrons.

### Limitations of this study.

The strengths of this study included the control of dietary intake before and during the inpatient procedures; matching of 2 sequential meals for food composition, taste, and form; provision of energy in the meals that was scaled to participants’ total daily energy needs; and the similarity between the groups with regard to sex and fasting blood chemistries. The primary limitation of the study relates to the nature of inpatient kinetic studies in which multiple blood samples are taken over a period of days, which limits the sample size. Also, to standardize the meals, we used stable isotopes of TAG containing only palmitate, and thus it would be important to determine whether similar quantities of unsaturated fatty acids are stored in healthy subjects (64). The inability to cleanly separate chylomicrons, remnants, and VLDL remains a key limitation in the field of postprandial lipemia. Limitations of the kinetic model are described in detail in Methods. The model could be applied to another in vivo data set, which could include meals of lesser or greater fat content. Importantly, each of the conclusions drawn from this in vivo human model can be mechanistically tested in animal models or in vitro.

In summary, we have developed and tested a model of human intestinal lipid metabolism whose structure is, in part, based on recent results from cell biology, rodent studies, and human isotopic research. Using an isotopic paradigm, we have found that less evening meal lipid was present in the plasma in IS subjects, suggesting that mechanisms exist in the intestine to buffer the impact of sequential meals on postprandial lipemia. This conclusion suggests that, in health, a physiological response to eating may be to slow lipid absorption throughout the day. In IR subjects, decreased efficiency of this process significantly contributes to postprandial hyperlipidemia, elevating lipemia at night and in the fasting state the next morning. Future studies should be performed to understand how meal timing and macronutrient composition of the evening meal might be optimized for those with insulin resistance to reduce postprandial hyperlipidemia and its associated cardiovascular disease risk.

## Methods

### Research subjects

Research subjects were recruited via advertisement. Eligible and interested subjects were recruited sequentially over a 2-year period. The study protocols were approved by the University of Missouri IRB (IRB 1208668) and subjects provided written, informed consent. Subjects were first screened by a phone interview, and preliminarily eligible subjects were then screened in person to rule out diabetes and obtain fasting biochemistry data. The average time between screening and admission 1 was 17 ± 13 days, and the time between admissions 1 and 2 was 19 ± 15 days. Recruitment criteria included nonsmoking status, age of 21–50 years, BMI 21–40 kg/m^2^, stable body weight over 3 months, and maintenance of normal activity patterns in men and premenopausal women. Potential subjects were screened to obtain fasting blood biochemistries and rule out diabetes. Subjects were excluded if they had known metabolic abnormalities or were taking medications known to affect lipid metabolism, were pregnant, consumed very high-fat (>50% of energy) or low-fat (<25% of energy) diets, performed shift work, exercised more than 3 days/week, consumed alcohol intake more than 140 g/week for men and 70 g/week for women, and had fasting plasma TAG greater than 300 mg/dL. For full inclusion and exclusion criteria, see ClinicalTrials.gov NCT02020343.

### Experimental procedures

#### Study design.

This study was designed as a 2-group comparison of the responses of IS and IR subjects to sequential meal consumption (lunch and dinner). Metabolic studies were conducted at the University of Missouri Clinical Research Center (CRC).

#### Procedures.

The study design is shown in Figure 1. In a separate clinical visit occurring before meal testing, subjects underwent FS-IVGTT to assess insulin sensitivity as described previously (44, 65). This method includes a bolus injection of glucose in the fasting state and measurements of glucose and the endogenous insulin response, followed by an injection of exogenous insulin at 20 minutes to assess accelerated insulin-mediated clearance of glucose. Glucose and insulin responses during the FS-IVGTT were analyzed using the minimal model (MINMOD) Millennium software (65). Using an established S_i_ cutoff of 2.5 (10^–4^ × min^–1^ per μU/mL), subjects were categorized as IS or IR (66). Compared with a labeled hyperinsulinemic-euglycemic clamp technique, which can be used to assess hepatic insulin sensitivity, the IVGTT was chosen to distinguish IS from IR subjects because it focuses on peripheral insulin sensitivity — a parameter that may also influence lipid clearance to the periphery. Within 3 weeks of the IVGTT, the patients participated in an overnight inpatient study to measure the kinetics of dietary fat absorption using a sequential meal paradigm. Subjects maintained habitual physical activity between the FS-IVGTT and the meal test. For 3 days before the inpatient meal test, subjects consumed a weight-maintaining diet formulated and produced by the University of Missouri Nutritional Center for Health metabolic kitchen. The present project does not meet the definition of a clinical trial. Supplemental Figure 1 describes the flow of subjects from recruitment to data analysis.

#### Tracer study protocol.

The morning of the inpatient study, subjects consumed, as outpatients, a low-fat breakfast with 18% of energy from fat, 12% from protein, and 70% from carbohydrate, providing 20% of daily energy needs. For the meal test, subjects reported to the CRC at 11:00 am for i.v. line placement, and then consumed 2 identical meals at 12:30 pm (lunch) and 6:00 pm (dinner). The goal of the meal formulations was to make the meals identical with respect to food content, composition, and form (cooking preparations), while at the same time presenting the foods as either a sandwich (with lettuce) or a salad (with bread) to reduce the likelihood of sensory-specific satiety (67). Lunch and dinner were each labeled with a different TAG stable isotope (^2^H_31_ glyceryl-tripalmitin and ^13^C_4_ glyceryl-tripalmitin), which was baked into a brownie fed for dessert as performed previously (68). On average, 1.5 g of TAG label was added to each brownie, resulting in a final meal TAG-palmitate enrichment of approximately 16%. The isotopes were fed in random order; 10 subjects consumed the ^2^H_31_ glyceryl-tripalmitin–containing brownie at lunch and the ^13^C_4_ glyceryl-tripalmitin–containing brownie at dinner; 6 subjects consumed meals in which the labels were switched. Meal consumption was completed within 15 minutes. Each meal provided 40% of total daily energy requirements based on the Harris-Benedict equation with an appropriate activity factor (69). The macronutrient composition of the meals remained constant in all subjects, providing 36% of energy from fat, 18% from protein, and 46% from carbohydrate (Table 2). Blood samples were taken through the i.v. line during the day and night. Immediately after blood collection, plasma was separated, a preservative cocktail was added (70), and samples were divided into aliquots for biochemical analysis (glucose, NEFA, etc.) and lipoprotein isolation. Energy expenditure was measured by indirect calorimetry during fasted (7:20 am) and fed (1:05 pm) conditions (Figure 1) and substrate oxidation calculated using the equation of Jequier (71). Body composition was measured by dual-energy x-ray absorptiometry (Hologic A, S/N 100158, analysis version 13.5.2 Auto Whole Body Fan Beam).

#### Outcomes.

The primary outcome of this study was to compare meal TAG absorption rates between IS and IR subjects.

#### Laboratory analysis.

Plasma TAG, NEFA, and glucose concentrations were measured enzymatically (kits 465-09791/461-09092, 991-34891, and 439-90901, respectively, Wako Diagnostics). Insulin (80-INSHU-E01.1, ALPCO Diagnostics), apoB48 (AKHB48; Shibayagi Co., Ltd., Biovendor LLC), and leptin (EZHL-80SK, MilliporeSigma) were measured by ELISA. Total TAG-rich lipoproteins (denoted TRL, and containing VLDL and chylomicrons) were isolated from plasma using fixed-angle ultracentrifugation at 40,000 RPM, or 1.3 × 10^8^*g*, for 20 hours in a 50.3 Ti rotor (Beckman Instruments) at 15°C (70), and samples were aliquoted for measurements of TAG and NEFA analysis. When TRL-TAG is hydrolyzed in the blood, some of the LPL-mediated release of chylomicron-TAG fatty acids escape tissue uptake and enter the albumin-bound NEFA pool (19). This process is termed spillover and was quantitated in the present study as described previously (72). TAGs from the TRL fraction and plasma NEFAs were separated by TLC and prepared for gas chromatography/mass spectrometry analysis as described (19, 70). The labeling patterns of TAGs and NEFAs were determined by measurement of isotopic enrichments on an Agilent 6890N GC coupled to a 5975 MS detector (Agilent Technologies). Isotope enrichments were determined by selective ion monitoring for *m/z* 270, 274 (derived from meal ^13^C_4_ glyceryl-tripalmitin), 300, and 301 (both derived from meal ^2^H_31_ glyceryl-tripalmitin) under electrical ionization using 4- to 5-point standard curves.

#### Systems biology modeling.

The model diagram in Supplemental Figure 2 represents transport processes and biochemical reactions shown to be involved in the trafficking of dietary fatty acids (62, 73–75). Kinetic analyses were undertaken to provide a quantitative test of the hypothesis represented by this diagram (76, 77). Testing in this way forced rejection of some model structures that were incapable of accounting for the experimental data. This physiologically based model includes 13 states, or species, as the term is used in the international standard for model exchange termed the systems biology markup language, SBML (78), and 16 processes (or reactions, SBML), including small intestinal transit delay modeled as 10 compartments in a series with uptake into enterocytes from each compartment. Wherever possible, processes were treated as linear mass action or simple Michaelis-Menten reactions, but this is a model that must account for the non–steady state produced by 2 successive substantial fat-containing meals (Table 2). Consequently, 5 of those processes are regulated by neuroendocrine signals: a) neurohumoral control of enterocyte cytosolic LD fusion to ER, b) insulin inhibition of enterocyte cytosolic LD hydrolysis, c) insulin inhibition of adipocyte LD hydrolysis, d) insulin activation of peripheral NEFA uptake, and e) leptin activation of direct chylomicron (particle) uptake (Supplemental Figure 2). Importantly, rate laws for these pathways are nonlinear because they are functions of measured hormone concentrations in each subject and thus are capable of accounting for total mass and tracer kinetics even when mass is decreasing while tracer is increasing as they are, for example, in the plasma NEFA pool. Model construction, simulation, testing, and parameter estimation were carried out in ProcessDB (Integrative Bioinformatics Inc.; ref. 79), which incorporates published methods for nonlinear, non–steady state tracer kinetics (80) and parameter optimization (81). The kinetic model structure is available in the supplemental materials as an SBML file, Supplemental Data 1.

#### Limitations of the kinetic model.

Limitations of the data propagate to limitations of the model. The most serious of these is the inability to measure tracer and TAG fatty acid mass in chylomicrons and VLDL separately. Second, our model cannot identify the precise metabolic processes responsible for the decreased appearance of dinner compared with lunch TAGs in plasma (e.g., oxidation versus particle uptake) and is limited by the lack of experimental evidence for direct chylomicron particle uptake independent of LPL. Additionally, the model cannot resolve tissue fates of fatty acids (adipose, skeletal muscle, cardiac muscle) or oxidation versus incorporation into glycerolipids.

### Quantification and statistical analysis

The absolute contribution of total dietary fat to TRL-TAG present in blood was calculated as described previously (70, 72). Briefly, the percentage of palmitate in the TRL-TAG fraction labeled with ^2^H_31_ glyceryl-tripalmitin or ^13^C_4_ glyceryl-tripalmitin (as determined by gas chromatography/mass spectrometry), was divided by the percentage enrichment of the meal to account for labeled and unlabeled palmitate in the meal. Meal composition and palmitate enrichments were calculated based on the precisely weighed amount of label added to the fat making up the brownie and the amount of total palmitate in the meals (Nutrition Data System for Research, NDSR 2014).

Results from the entire 21-hour duration of data collection were divided into 3 segments that included a post-lunch phase between 12:30 pm and 6:00 pm, a post-dinner phase between 6:00 pm and 11:00 pm, and a nocturnal phase between 11:00 pm and 7:00 am. For all biochemical and isotopic data (presented as absolute quantities of label in the TRL-TAG fraction in the figures), the integrated AUC was calculated for comparison between the time segments. After 12:30 pm, the lunch label reached a peak and then began to decay. The SME was considered in 2 ways. First, SME was defined as the abrupt increase in the lunch label appearing within 1.5 hours after the onset of dinner and was calculated as the lunch area above that which would have been expected in TRL following a modeled decay of the lunch label that would occur had a second meal (dinner) not been consumed (82, 83). Second, the peak was calculated based on the model (Table 3, row 5).

The duration of data collection during and after the meal tests was divided into 3 segments that included a post-lunch phase between 12:00 pm and 6:00 pm, a post-dinner phase between 6:00 pm and 11:00 pm, and a nocturnal phase between 11:00 pm and 7:00 am. Concentrations of plasma TAG, TRL-TAG, and TRL-apoB48 over 3–4 hours after lunch or dinner were analyzed as incremental AUC relative to the baseline value occurring before lunch (12:20 pm). The absolute contribution of total dietary fat to TRL-TAG present in blood was calculated as follows. At each time that the TRL-TAG was isolated, the percentage of palmitate in the TRL-TAG fraction labeled with ^2^H_31_ glyceryl-tripalmitin or ^13^C_4_ glyceryl-tripalmitin (as determined by GC/MS) was divided by the percentage enrichment of the meal. The percentage of label in the TRL-TAG pool was then multiplied by the TAG concentration in this fraction to determine the total TRL-TAG derived from the meals, including labeled and unlabeled fatty acids (*Equation 1*). This calculation assumes that the labeled TAG palmitate is absorbed in a manner similar to TAG carrying other fatty acids. The composition and palmitate enrichments of the meals were calculated based on the precisely weighed amount of label added to the fat making up the brownie and the calculated amount of total palmitate in the meals (determined by using the Nutrition Data System for Research, NDSR 2014).

#### Equation 1.

Total TRL-TAG derived from meals = (%M30 + M31 or %M4 in TRL-TAG/isotopic enrichment of the meal [%]) × TRL-TAG concentration (mmol/L). To compare with the postprandial literature, data are presented as TRL-TAG concentrations derived from meals. However, for the calculations of labeled and unlabeled palmitate in TRL-TAG that were used in the model development and testing described below, the TAG concentration in this fraction was multiplied by 3 and then by the percentage of palmitate in TRL-TAG fatty acids that was palmitate as determined by gas chromatography. This concentration was multiplied by the percentage of palmitate that was labeled with ^2^H_31_ glyceryl-tripalmitin or ^13^C_4_ glyceryl-tripalmitin (*Equation 2*).

#### Equation 2.

TRL-TAG palmitate derived from meals = TRL-TAG concentration (mmol/L × 3) × %TAG fatty acids that are palmitate × %M30 + M31 or %M4. The labeled and unlabeled palmitate from diet that was present in the plasma NEFA pool was calculated by multiplying plasma NEFA concentration by the percentage of palmitate in NEFA fatty acids that was palmitate. This concentration was multiplied by the percentage of palmitate that was labeled with ^2^H_31_ glyceryl-tripalmitin or ^13^C_4_ glyceryl-tripalmitin (*Equation 3*).

#### Equation 3.

NEFA palmitate derived from meals = NEFA concentration (μmol/L) × %NEFA fatty acids that are palmitate × %M30 + M31 or %M4.

#### Equation 4.

The SME was calculated as the absolute AUC above that which would be expected by the decay of the lunch label if the second meal (dinner) had not elicited the abrupt absorption of the lunch meal TAGs. The lunch TAGs used for these calculations represent the labeled and unlabeled TAGs derived from lunch.

#### Statistics.

Statistical analyses were performed using GraphPad Prism (version 9). IS and IR groups were compared by unpaired *t* test (data presented as mean ± SD). Data varying by time between groups were analyzed by repeated-measures 2-way ANOVA (and reported as mean ± SEM) with post hoc tests by Sidak’s multiple-comparison test. Differences within the group were analyzed by paired, 2-tailed *t* tests or 1-way repeated-measures ANOVA, as followed by Tukey’s post hoc test to locate significant mean differences where appropriate. All variables were checked for normal distribution, and nonparametric analyses (Mann-Whitney tests) were used for variables with skewed distribution as noted. Kinetic measures of postprandial metabolism (shown in Table 3) were adjusted for multiple comparisons by controlling the FDR at 0.05. Relationships among rates of appearance, kinetic parameters, and selected metabolic variables were evaluated by linear regression analysis. All statistical tests were 2 sided, with a *P* value of 0.05 considered statistically significant and *P* less than 0.10 indicating a trend.

On the basis of the interindividual variability in TRL-TAG in the fed condition assessed in IS and IR individuals and reported by others previously (7), we estimated that 8 individuals per group would be needed to detect between-group differences in TRL-TAG after meals of 0.5 mmol/L using a 2-sided test with a β value of 0.95 and an α value of 0.05. These computations were performed using G*Power 3.1.9.7 (84).

#### Study approval.

Individuals provided written, informed consent before participating in this study, which was approved by University of Missouri IRB.

## Author contributions

MJS collected, analyzed, and interpreted the data and wrote the manuscript; QH aided in data collection and edited the manuscript; CMMA provided medical oversight and contributed to manuscript writing; RDP designed the modeling approach, performed the modeling, and contributed to data analysis and interpretation and to manuscript writing; and EJP designed the study, analyzed and interpreted the data, and wrote the manuscript.

## Supplementary Material

Supplemental data

Systems Biology Markup Language file 1

## Figures and Tables

**Table 3:**
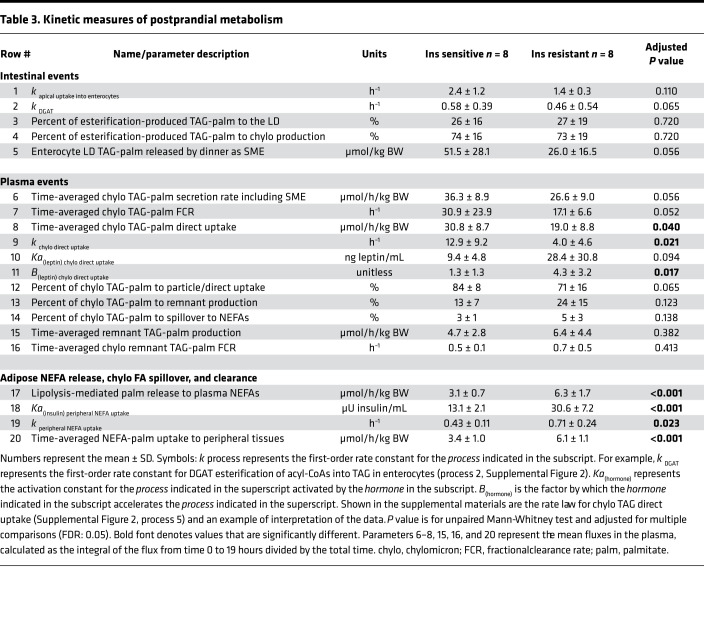
Kinetic measures of postprandial metabolism

**Table 1:**
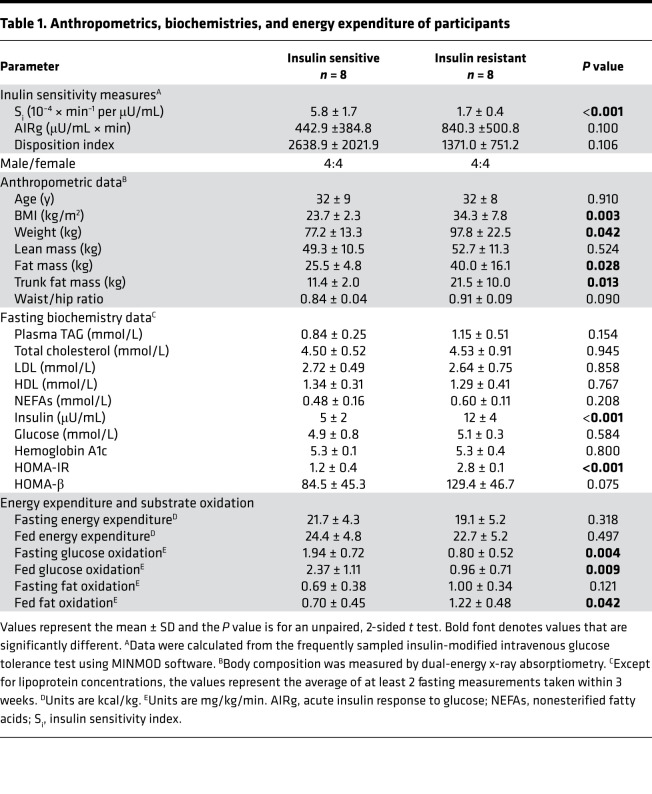
Anthropometrics, biochemistries, and energy expenditure of participants

**Table 2:**
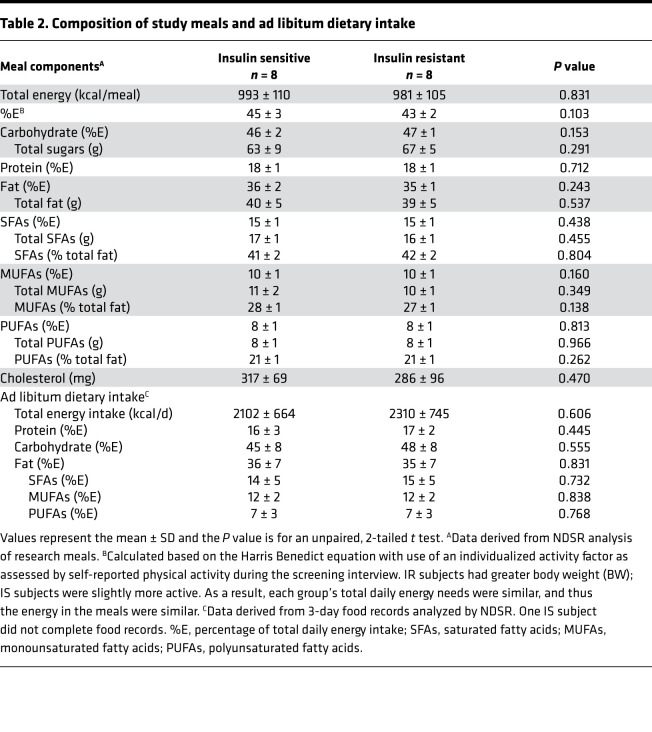
Composition of study meals and ad libitum dietary intake

## Version 1 08/09/2021

Electronic publication
