# Supplementary material for: Human intestinal lipid storage through sequential meals reveals faster dinner appearance is associated with hyperlipidemia
Source: JCI Insight. 2021 Aug 9;6(15):e148378. doi: 10.1172/jci.insight.148378 (PMC8489663; doi:10.1172/jci.insight.148378)
Supplement: Supplemental data [file jciinsight-6-148378-s010.pdf]

SUPPLEMENTAL FIGURES

Supplementary figure 1. Flow of subjects from recruitment to data analysis

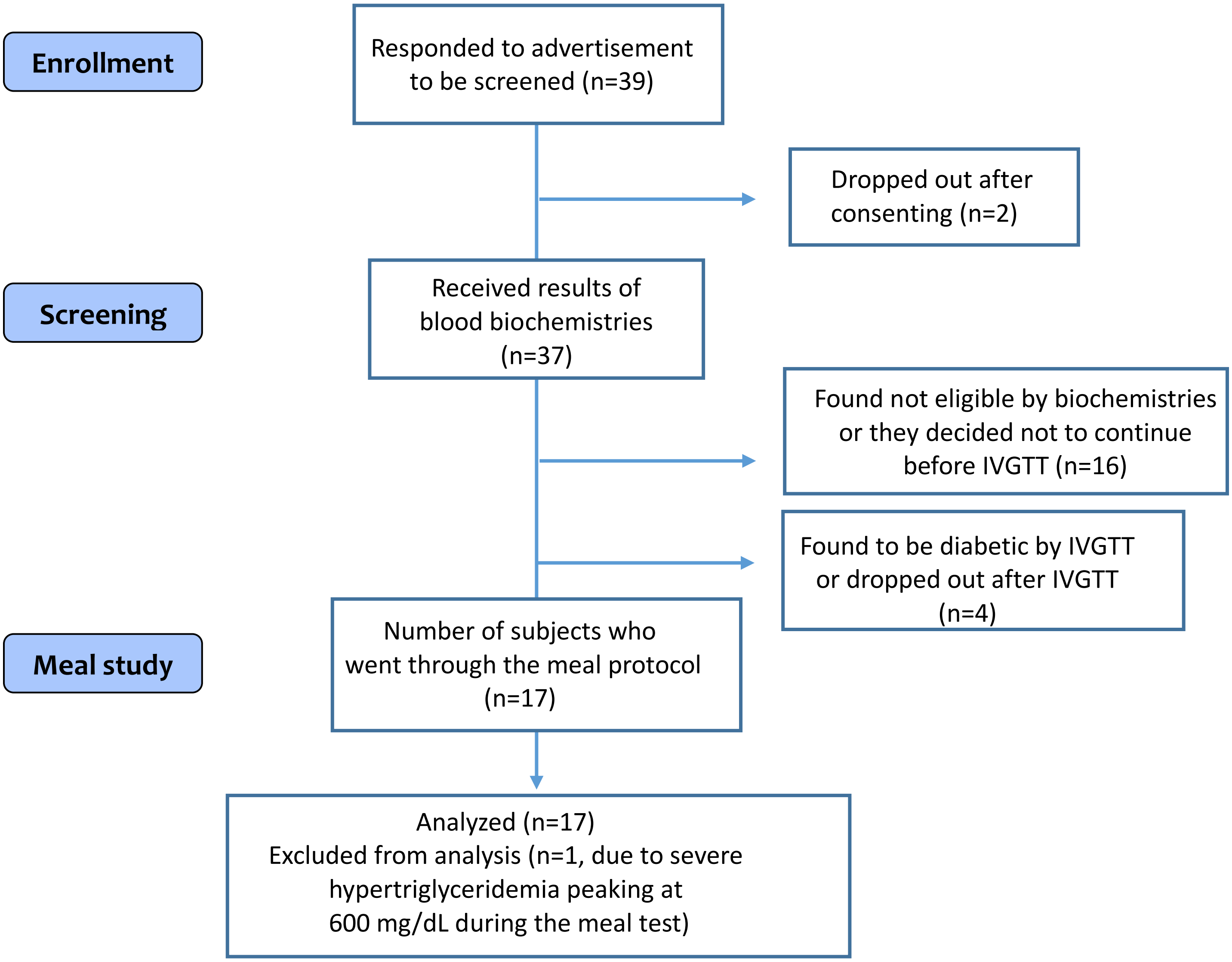

Data analysis

Final data analyzed (n=16)					
ID #	Insulin sensitive		ID #	Insulin resistant	
1	Female	White	3	Male	Asian
2	Male	Asian	4	Male	Asian
5	Female	White	6	Female	White
10	Male	White	7	Female	AA
14	Female	White	8	Male	African
15	Male	White	9	Female	Hispanic
16	Female	White	11	Female	White
21	Male	White	13	Male	White

Supplementary figure 2. Known biochemical processes of lipid absorption included in the nonlinear, non-steady state model

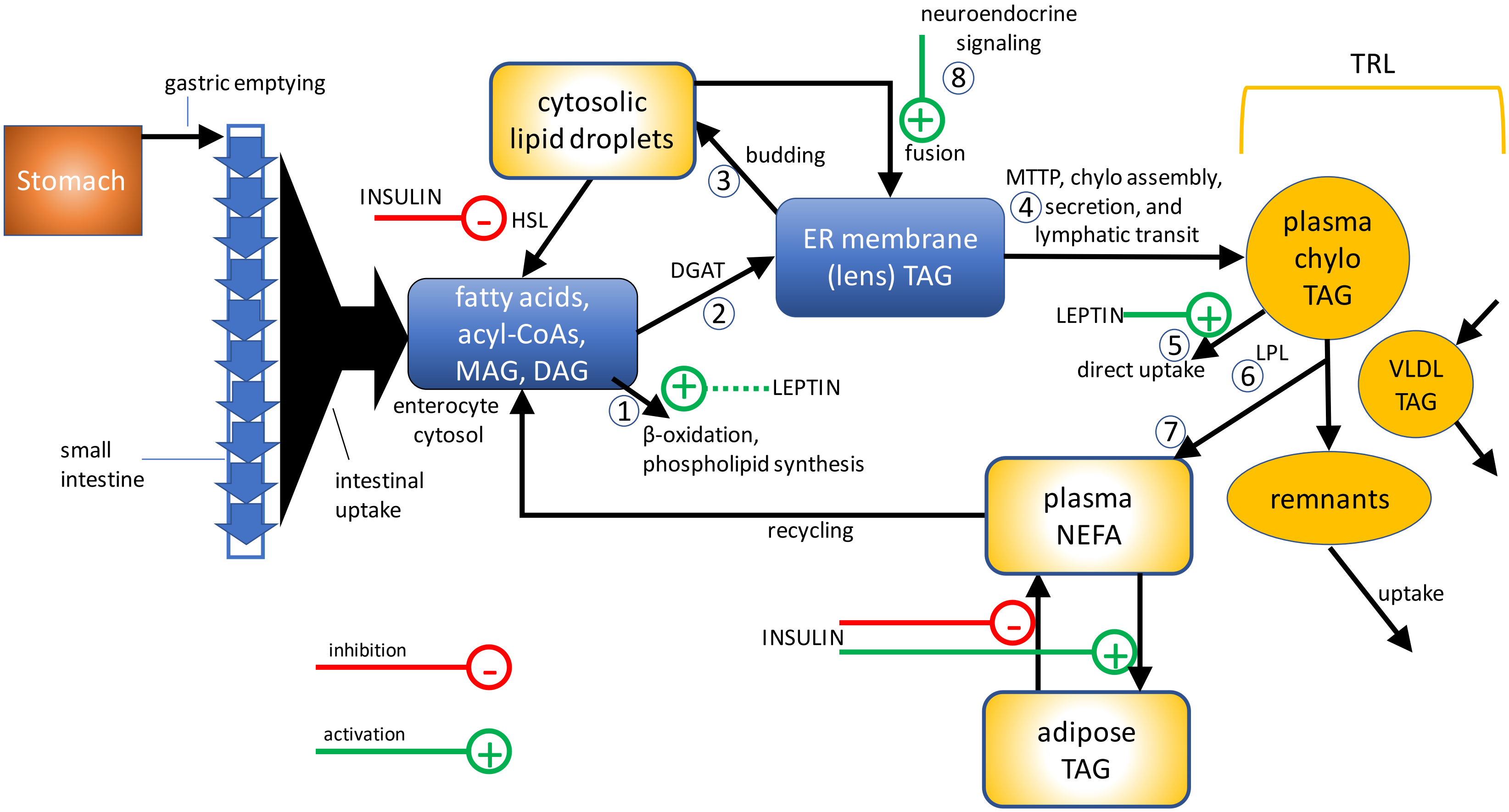

After consumption, delivery of meal lipid is regulated by gastric emptying, intestinal transit, enterocytic processes of lipid transport, biochemistry, and storage. Meal-derived cellular fatty acids may be routed to β-oxidation or to the synthesis of other complex lipids such as phospholipids - denoted **Process 1** in the figure. Meal fatty acids also may be immediately resynthesized into TAG in the ER membrane (**Process 2**). Farese et al have used the word 'lens' to describe ER TAG residing between the ER membrane leaflets (1).

Lens-TAG can be delivered to existing cytosolic LD or undergo budding to produce new LDs (**Process 3**) or can be added to primordial lipoprotein particles as they mature through the ER and Golgi (**Process 4**). This process includes addition of lipid through action of the enzyme microsomal TAG transfer protein (MTTP), movement of the particle through the Golgi, followed by secretion into the basolateral spaces and lymph. Once in plasma, meal lipid carried in chylomicrons are subject to removal by particle/direct uptake (**Process 5**) or clearance by lipoprotein lipase (LPL, **Process 6**).

Lipolysis of chylomicrons proceeds at a rate that exceeds tissue uptake and some meal-derived fatty acids enter the plasma NEFA pool. This process is called dietary spillover (**Process 7**). As reported (2), a small fraction of plasma NEFA is recycled to enterocytes via the basolateral membranes. Hormonal control of these processes is also represented in the figure. If the meal contains carbohydrate (as it did here), increases in insulin concentration inhibit adipose lipolysis reducing plasma NEFA concentration. This occurs at the same time as spillover enters the NEFA pool. Insulin also stimulates uptake of dietary fatty acids into adipose (and other tissues, (3). Hormone sensitive lipolysis (HSL) on the figure represents the system of a series of enzymes that lipolyze cellular LD. These processes are inhibited by insulin. Leptin is hypothesized to increase peripheral direct uptake of chylomicrons or diversion of enterocyte fatty acids to oxidation or phospholipid synthesis. Lastly, hypothesized taste/neuroendocrine control causing direct release of stored lipid is represented in **Process 8**. This process aims to account for published SME observations (4, 5) as well as our current data.
The final parameters and data are presented in the manuscript's table 3. In that table. the rate law for chylo TAG direct uptake (**supplemental figure 2 process 5**) is $k_{chyloDirectUptake} T_{pl}^{chylo} \left[1 + B_{leptin}^{chyloDirectUptake} \frac{L_{pl}}{K_{a(leptin)}^{chyloDirectUptake}} \right]$, where $T^{chylo}{}_{pl}$ is TAG-palmitate in chylomicrons in plasma (*pl*), and L_{pl} is leptin in plasma. Analysis of meal transients resulted in time-varying fluxes, which were integrated and normalized to the total time of the experiment to obtain time-averaged fluxes and time averaged fractional catabolic rates.

<u>An example of how the literature is used to develop the model</u>: Esterification activity, as estimated by DGAT activity: That DGAT (**Table 3, row 2**) is rate-limiting for delivery of meal fat to plasma is not a novel idea (6) but the importance of this fact in interpreting and quantifying the SME is novel. We approached this problem by considering the rates of processes between stomach and

enterocyte and between enterocyte and blood. Historical views that lymph transport is slow compared to capillary blood flow (~1 mm/s) have proven inconsistent with direct measurement. Lymphatic flow velocity (in rats) is ~3 mm/s (7). Hence, the extrapolated time required to travel the ~300 mm from lacteal to subclavian vein (in humans) is ~100 s. In contrast, our estimate (based on the need to account for labeled palmitate accumulation in plasma) of the esterification-limited palmitate residence time in enterocytes is ~2h.

An example of interpretation of the data in Table 3: **Rows 6-8** present the mean fluxes of lipids in the plasma, calculated as the integrals of the time-varying fluxes from time 0h to 19h divided by the total time. Chylomicron TAG-palmitate secretion rates (**row 6**) were 27% higher in insulin sensitive subjects compared to insulin resistant subjects (*P*=0.056), while the fractional catabolic rate (FCR, **row 7**) was 45% higher in insulin sensitive (*P*=0.052). The loss of chylomicron-TAG from plasma via a very fast system (**row 8**, denoted direct uptake) was over 1.6-fold faster (*P*=0.040), while as shown in **row 9**, the removal rate was 3-fold higher. Specifically, insulin sensitive subjects exhibited 12.9±0.3 pools/h traversing this pathway compared to 4.0±4.6 pools/h in insulin resistant subjects (*P*=0.021). The influence of a natural circadian increase in plasma leptin concentrations (**figure 6A-C**) was also tested in the model. The coefficient for leptin activation of chylomicron-TAG removal from plasma (**row 10**) was 9.4±4.8 ng/mL in insulin sensitive and 28.4±30.8 in insulin resistant subjects (*P*=0.094); a similar factor (**row 11**) by which leptin facilitates chylomicron-TAG uptake was 4-fold higher.

Supplementary figure 3. Relationships between kinetic parameters, S_i, and meal AUCs

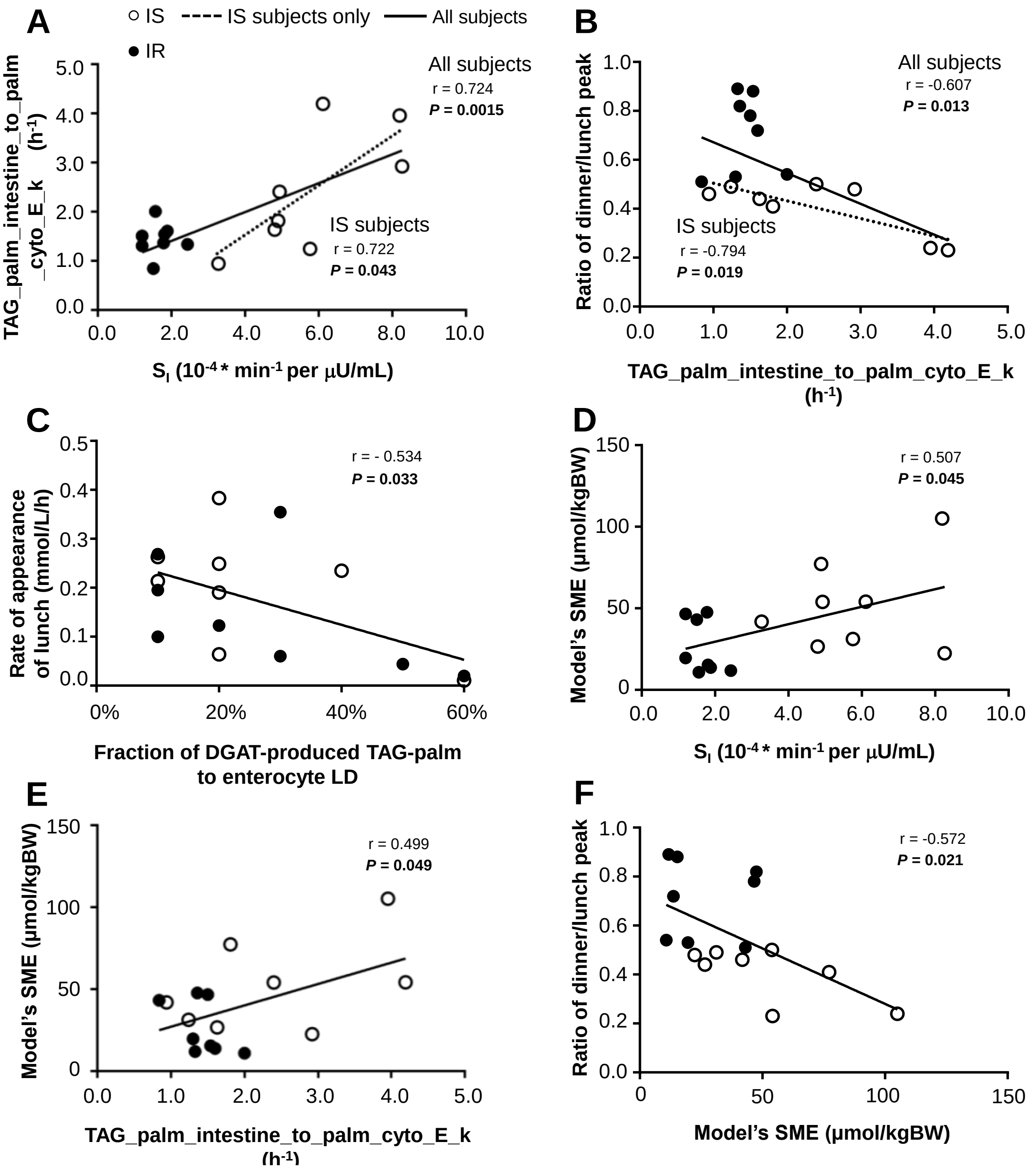

(**A**) Relationships between the insulin sensitivity index (Si) and the model's calculated fractional meal-TAG absorption per hour from the intestinal lumen into the enterocyte; **(B)** between fractional meal-TAG absorption per hour from the intestinal lumen into the enterocyte and the ratio of the plasma dinner peak height / the lunch peak height; **(C)** between the fraction of lipid moving from intracellular esterification pools to enterocyte LD and the rate of appearance of lunch in plasma, **(D)** between S_i and the model's calculated enterocyte LD TAG released by dinner as SME **(E)** between fractional meal-TAG absorption per hour from the intestinal lumen into the enterocyte and the model's calculated enterocyte LD TAG released by dinner as SME, and **(F)** the model's calculated enterocyte LD TAG released by dinner as SME and the ratio of the plasma dinner peak height / the lunch peak height. Pearson correlations for all subjects combined denoted with a solid line and for IS subjects only with a dotted line. Only *P* values of correlations for all subjects combined are shown.

REFERENCES

1. Gluchowski NL, Becuwe M, Walther TC, and Farese RV, Jr. Lipid droplets and liver disease: from basic biology to clinical implications. *Nat Rev Gastroenterol Hepatol.* 2017;14(6):343-55.
2. Storch J, Zhou YX, and Lagakos WS. Metabolism of apical versus basolateral sn-2-monoacylglycerol and fatty acids in rodent small intestine. *J Lipid Res.* 2008;49(8):1762-9.
3. Ramos-Roman MA, Lapidot SA, Phair RD, and Parks EJ. Insulin activation of plasma nonesterified fatty acid uptake in metabolic syndrome. *Arterioscler Thromb Vasc Biol.* 2012;32(8):1799-808.
4. Chavez-Jauregui RN, Mattes RD, and Parks EJ. Dynamics of fat absorption and effect of sham feeding on postprandial lipema. *Gastroenterology.* 2010;139(5):1538-48.
5. Mattes RD. Oral exposure to butter, but not fat replacers elevates postprandial triacylglycerol concentration in humans. *J Nutr.* 2001;131(5):1491-6.
6. Yen CL, Stone SJ, Koliwad S, Harris C, and Farese RV, Jr. Thematic review series: glycerolipids. DGAT enzymes and triacylglycerol biosynthesis. *J Lipid Res.* 2008;49(11):2283-301.
7. Dixon JB, Zawieja DC, Gashev AA, and Coté GL. Measuring microlymphatic flow using fast video microscopy. *Journal of biomedical optics.* 2005;10(6):064016.
